# Interactive associations of daily music engagement and nutritional behavior with psychological performance in competitive athletes: a 14-day naturalistic diary study

**DOI:** 10.3389/fnut.2026.1921086

**Published:** 2026-08-24

**Authors:** Dekun Peng

**Affiliations:** Hunan Vocational College of Electronic and Technology, Changsha, Hunan, China

**Keywords:** athlete, competitive sports, music engagement, naturalistic digital diary, nutrition

## Abstract

**Background:**

Music engagement and nutrition are each linked to athletes’ mental states, yet their combined, day-to-day influence under real training conditions remains poorly understood.

**Objective:**

To determine whether daily music engagement and nutritional behavior are independently and interactively associated with psychological performance in competitive athletes.

**Methods:**

In a 14-day naturalistic digital diary study, 180 competitive athletes (≥18 years) from university teams and sports clubs reported daily music engagement, nutritional behavior, training load, sleep quality, and psychological performance. The primary analyses used multilevel mixed-effects models to separate within-person from between-person variation and to test the music × nutrition interaction. The final reported dataset comprised 2,184 athlete-day observations from 156 athletes; the handling of excluded participants and missing records is described in the Methods.

**Results:**

Within-person increases in music engagement (*β* = 0.19, *p* < 0.001) and nutritional behavior (*β* = 0.24, *p* < 0.001) were each independently associated with higher same-day psychological performance. The music × nutrition interaction was positive (*β* = 0.11, *p* = 0.003), indicating that the association between music engagement and psychological performance was stronger on days characterized by more favorable nutritional behavior.

**Conclusion:**

More favorable daily music engagement and nutritional behavior were jointly associated with greater psychological readiness. Because the design was observational and concurrent, the findings should be interpreted as within-person covariation and require confirmation in prospectively powered experimental studies.

## 1 Introduction

Psychological preparedness is increasingly recognized as an important component of competitive sport. Mood, focus, motivation, perceived stress, and recovery are associated with how athletes train, compete, and adapt to training load, and day-to-day variation in these states may have meaningful performance implications (1–3). Accordingly, identifying routine behaviors associated with athletes’ psychological readiness is relevant to sport scientists and practitioners.

Music engagement is one such behavior. Research in exercise and sport has associated music with more favorable affect (4), motivation (5), attentional regulation (6), perceived exertion (7), and selected recovery or performance outcomes (8). Athletes may use music before training or competition to regulate arousal and anxiety (9) and after activity to facilitate relaxation (10). However, much of the evidence derives from laboratory experiments (11) or single-time-point surveys (12) and therefore provides limited information about within-athlete variation across ordinary training days.

Nutritional behavior is also associated with psychological and physical functioning (13). Meal regularity, hydration (14), carbohydrate (15) and protein availability (16), and overall dietary quality (17) may coincide with mood, cognition, fatigue, recovery, and training readiness (15). Nevertheless, much of the available research compares athletes with one another rather than examining whether an athlete’s daily nutritional behavior covaries with that athlete’s psychological state (18, 19).

A notable gap therefore persists at the intersection of these behaviors. Music engagement and nutritional behavior are generally examined in separate research literatures, leaving unclear whether they show independent associations with psychological performance or a statistical interaction when assessed within the same athlete. A positive interaction would indicate that the association between one behavior and psychological performance differs according to the daily level of the other behavior; it would not, by itself, establish a causal or biological synergy.

Single-time-point questionnaires cannot distinguish stable differences between athletes from processes that vary within athletes across training, recovery, and competition-related contexts (20). Intensive longitudinal diary designs address this limitation by obtaining repeated observations in natural settings and permitting multilevel analysis of within-person and between-person variability (21, 22).

The present study used a 14-day naturalistic digital diary design to examine routine music engagement, nutritional behavior, and psychological performance in competitive athletes. The objectives were to determine whether daily music engagement and nutritional behavior were independently associated with same-day psychological performance and whether a music × nutrition interaction was present. No music, dietary, supplement, or psychological intervention was introduced.

## 2 Methods

### 2.1 Study design

A naturalistic digital diary design was used to examine day-to-day associations of music engagement and nutritional behavior with psychological performance in competitive athletes. The study was observational and non-interventional and was conducted in participants’ usual training and competition environments. The researchers did not assign a music program, diet plan, supplement, therapy, experimental task, or group allocation. Because participants were not prospectively assigned to a health-related intervention, clinical-trial registration was not applicable. Repeated athlete-day observations were nested within individuals, allowing within-person and between-person variation to be modeled separately.

### 2.2 Participants and setting

Competitive athletes were recruited from university sports teams and sports clubs. Eligible participants were aged 18 years or older and engaged in regular training and competition. Team-sport and individual-sport athletes were included. Eligibility was assessed against these prespecified inclusion and exclusion criteria before enrollment. Athletes receiving formal music therapy, prescribed dietary treatment, clinical psychological treatment, or medically supervised nutrition during data collection were excluded. No additional exclusion criteria were applied.

### 2.3 Sampling and sample size

A convenience sampling method was used. Coaches, team managers, and sport administrators distributed the study invitation to potentially eligible athletes, who participated voluntarily. A total of 180 athletes entered the 14-day diary phase, corresponding to 2,520 potential athlete-day observations. The final analytical dataset comprised 156 athletes at Level 2 and 2,184 athlete-day observations at Level 1. Repeated observations from the same athlete were correlated and were not treated as independent participants. No formal *a priori* power analysis was used to determine recruitment; recruitment was based on the number of eligible athletes accessible during the predefined data-collection period. Accordingly, the interaction estimate is interpreted using its standardized coefficient, standard error, and 95% confidence interval rather than being described as having “large power.” Sample-size adequacy in a two-level longitudinal model depends on the numbers of athletes and repeated observations, the intraclass correlation, the random-effects structure, and the effect size of interest (23, 36–38).

### 2.4 Study duration

Data were recorded over 14 consecutive days. This period was selected to capture short-term variation in music engagement, nutritional behavior, training load, recovery, mood, stress, motivation, and psychological readiness while limiting participant burden and potential decline in diary completion.

### 2.5 Data collection procedure

Data were gathered in two phases. First, participants completed a baseline questionnaire covering demographic characteristics, sport type, competitive experience, training frequency, general music-listening habits, usual dietary behavior, and baseline psychological characteristics. These baseline variables were used to describe the sample and provide covariates for the multilevel analyses. Second, participants completed a brief digital diary once daily for 14 consecutive days using a mobile phone or online form; completion required approximately 3–5 min. Each diary assessed the participant’s music engagement, nutritional behavior, training load, sleep quality, and psychological state for that day. Participants were instructed to report their usual behavior and were not asked to modify music or dietary practices. The diary was designed for daily completion during the observation period; no experimental exposure, dietary prescription, supplement, or formal compliance incentive was provided.

### 2.6 Measures

Daily music engagement was assessed using a brief author-developed diary composite covering whether music was used, listening duration and timing, listening context (e.g., before or after training, before competition, during recovery, or during personal activities), and perceived functions including motivation, relaxation, mood regulation, stress reduction, concentration, and recovery. Daily nutritional behavior was assessed using items covering meal regularity, breakfast consumption, hydration, fruit and vegetable intake, protein-rich foods, carbohydrate intake around training, omega-3-rich foods, and perceived nutritional adequacy. Daily psychological performance was assessed using items addressing mood, motivation, mental focus, perceived stress, pre-training or pre-competition anxiety, fatigue, perceived recovery, and overall psychological readiness. Negatively oriented psychological items, including stress, anxiety, and fatigue, were reverse-coded before calculation of the composite so that higher scores consistently represented more favorable psychological performance. For each domain, available item scores were averaged to form a daily composite score; higher music-engagement scores indicated greater engagement, higher nutritional-behavior scores indicated more favorable daily nutritional practices, and higher psychological-performance scores indicated a more favorable psychological state. Covariates included age, sex, sport type, competitive level, training frequency, daily training load, and sleep quality. Because the diary composites were developed for this study, their within-person and between-person psychometric properties require independent validation and are acknowledged as a limitation. The music × nutrition interaction was operationalized as the product of person-mean-centered daily music-engagement and nutritional-behavior scores. No separate summed “synergy index” was used in the primary model.

### 2.7 Missing-data assessment and handling

Participant-level attrition and diary-level missingness were evaluated separately. Of the 180 athletes entering the diary phase, 24 were excluded because they did not provide complete 14-day diary records that satisfied the prespecified completeness requirement. The remaining 156 athletes contributed 2,184 complete athlete-day observations (156 athletes × 14 days). Thus, the reduction from 2,520 potential records to 2,184 analyzed records reflects the exclusion of the complete 14-day series for 24 athletes rather than intermittent missing values within the retained analytical sample.

Because the primary analytical file contained complete 14-day records for the 156 retained athletes, the reported models were based on a complete-case sample. The study did not establish that the excluded records were missing completely at random. Consequently, systematic differences between retained and excluded athletes cannot be ruled out, and the potential for attrition-related selection bias is acknowledged in the Limitations. The missing-data mechanism is therefore treated cautiously rather than assumed to be MCAR.

The primary analysis used the complete-case dataset of 2,184 athlete-day observations from 156 athletes. No values were imputed. This approach was used because the study applied a prespecified requirement for complete 14-day diary records and the final analytical file contained no intermittent missing values for the variables included in the reported models. However, complete-case analysis may reduce precision and may introduce selection bias when exclusion is related to observed or unobserved participant characteristics. The results should therefore be interpreted cautiously, and future studies should retain all available diary observations through maximum-likelihood estimation (24) and use multilevel multiple imputation when predictors or covariates are missing.

### 2.8 Statistical analysis

Daily observations (Level 1) were nested within athletes (Level 2); therefore, multilevel linear mixed-effects models were used to separate within-person from between-person variation (25, 26). Continuous variables were standardized, and daily music engagement and nutritional behavior were person-mean-centered to isolate within-athlete deviations from each athlete’s own mean. A random intercept for athlete accounted for differences in baseline psychological performance. The primary model included within-person music engagement, within-person nutritional behavior, their product term, daily training load, sleep quality, and the prespecified demographic and sport-related covariates. Standardized regression coefficients (*β*), standard errors, 95% confidence intervals, and two-sided *p* values are reported.

Daily psychological performance = music engagement + nutritional behavior + (music × nutrition) + training load + sleep quality + demographic and sport covariates + (random intercept | athlete).

A positive interaction term indicated that the within-person association between music engagement and psychological performance varied according to daily nutritional behavior. This statistical interaction was interpreted as evidence of moderation rather than proof of a causal or biological synergy. Model assumptions were evaluated conceptually through the mixed-model specification, and model fit was compared using the Akaike and Bayesian information criteria. Standardized coefficients, standard errors, 95% confidence intervals, and *p* values are reported for all fixed effects.

### 2.9 Ethical considerations

This study involving questionnaire and digital-diary data from competitive athletes was reviewed and approved by the Ethics Committee of Hunan Electronic Science and Technology Vocational College (Approval No. HESTVC-EC-2026-496; approval date: 13 March 2026). The research was non-interventional and examined participants’ music engagement, nutritional behavior, and psychological performance. Before participation, all participants received relevant study information and provided written informed consent. Participation was voluntary, and participants were permitted to withdraw at any time without penalty. All personal information and research data were kept strictly confidential and used exclusively for academic research purposes. Because participants were not prospectively assigned to a health-related intervention, clinical-trial registration was not applicable.

## 3 Results

### 3.1 Preliminary analyses and data structure

**Figure 1:**
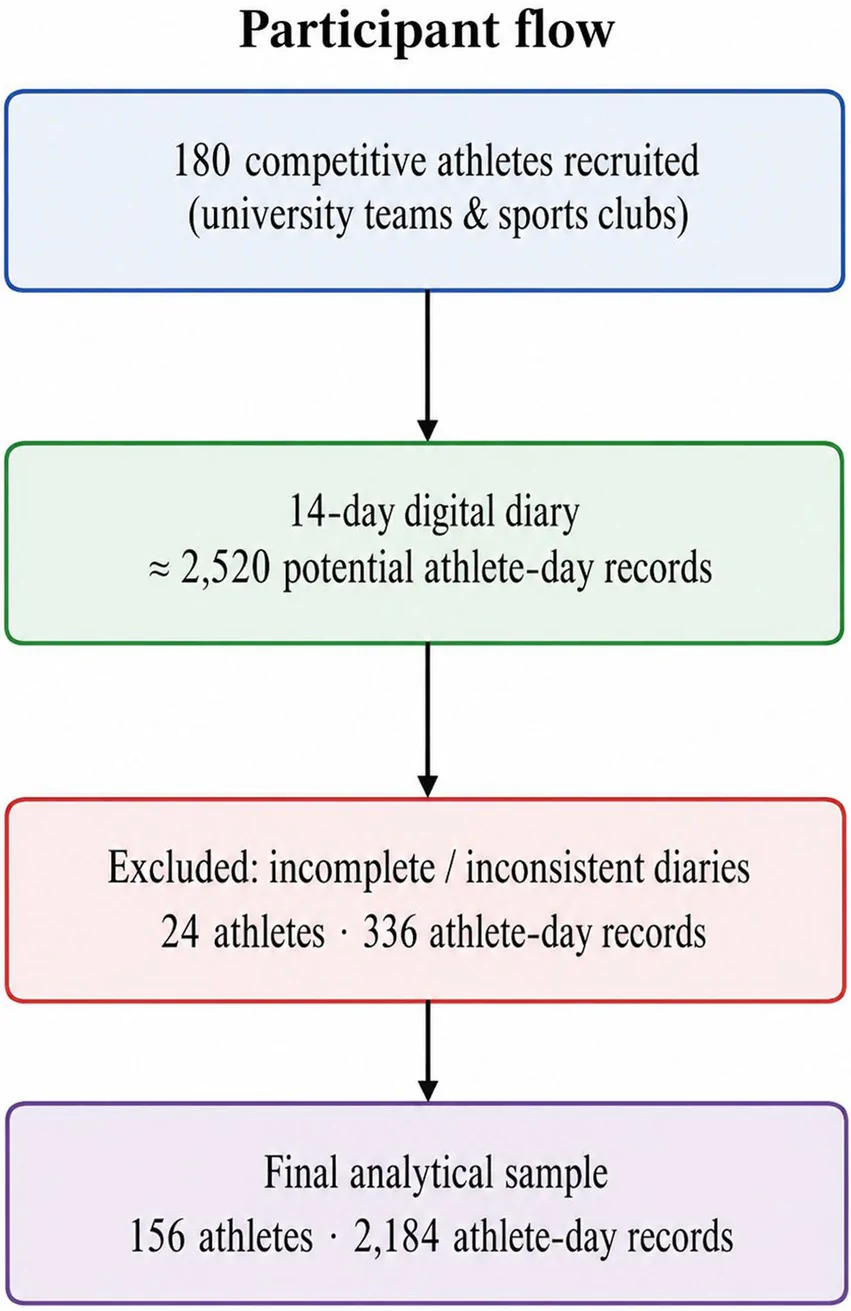
Participant and athlete-day record flow.

**Figure 2:**
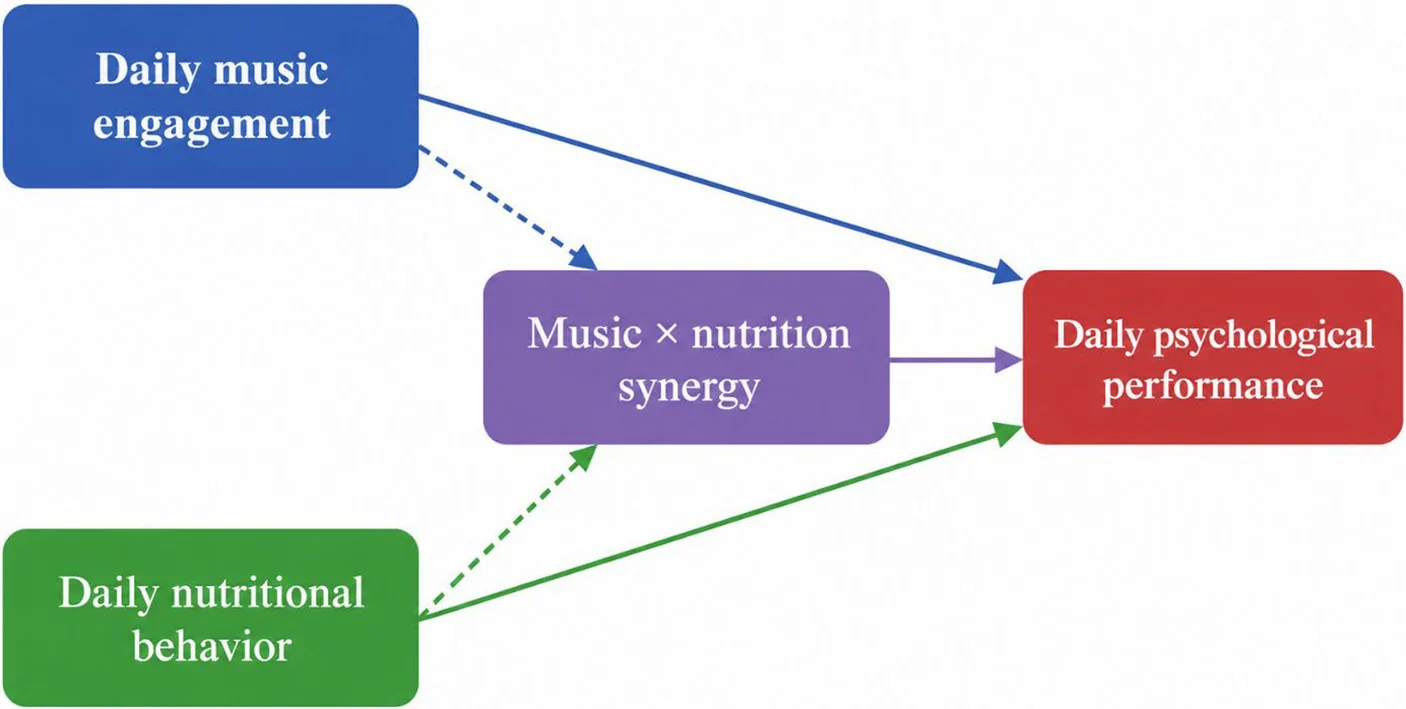
Conceptual framework. Hypothesized within-person model in which daily music engagement and nutritional behavior are independently associated with daily psychological performance and interact statistically after adjustment for training load, sleep quality, and demographic and sport-related covariates.

A total of 180 athletes entered the diary phase, of whom 156 (86.7%) met the complete-record criterion and were included in the analytical sample. The 2,184 analyzed athlete-day records represented 86.7% of the 2,520 potential records (Figure 1). This percentage is reported as record availability rather than daily compliance. The analytical sample included team-sport and individual-sport athletes across several competitive levels (Table 1). An unconditional multilevel model was estimated to partition between-athlete and within-athlete variance in daily psychological performance. Formal comparisons between retained and excluded athletes were not available in the source analytical report; therefore, systematic attrition cannot be excluded and is acknowledged as a limitation (Figure 2).

**Table 1:** Sample characteristics and record availability.

Characteristic	Value
Participants entering diary phase, *n*	180
Participants retained in reported analysis, *n* (%)	156 (86.7)
Athlete-day records analyzed, *n*/possible (%)	2,184/2,520 (86.7)
Age, years, mean (SD)	21.4 (2.8)
Sex, *n* (% female)	68 (43.6)
Team sport, *n* (%)	92 (59.0)
Individual sport, *n* (%)	64 (41.0)
Competitive level, *n* (%)	Regional: 91 (58.3); national: 50 (32.1); university/club: 15 (9.6)
Training experience, years, mean (SD)	5.8 (2.7)
Weekly training frequency, mean (SD)	5.1 (1.4) sessions/week
Baseline psychological-performance score, mean (SD)	3.58 (0.61)

**Figure 3:**
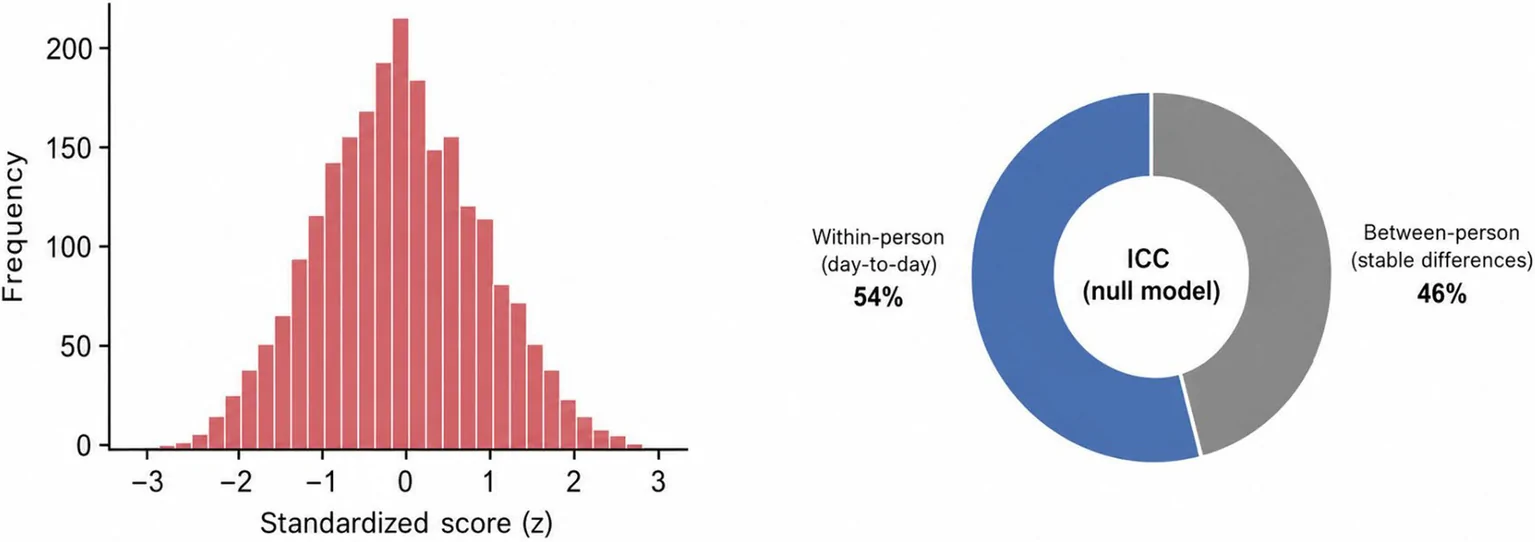
Distribution and variance partition of daily psychological performance. The left panel presents the reported distribution of standardized daily psychological-performance scores. The right panel presents the variance partition from the unconditional multilevel model, with approximately 45% of variance between athletes and 55% within athletes.

**Figure 4:**
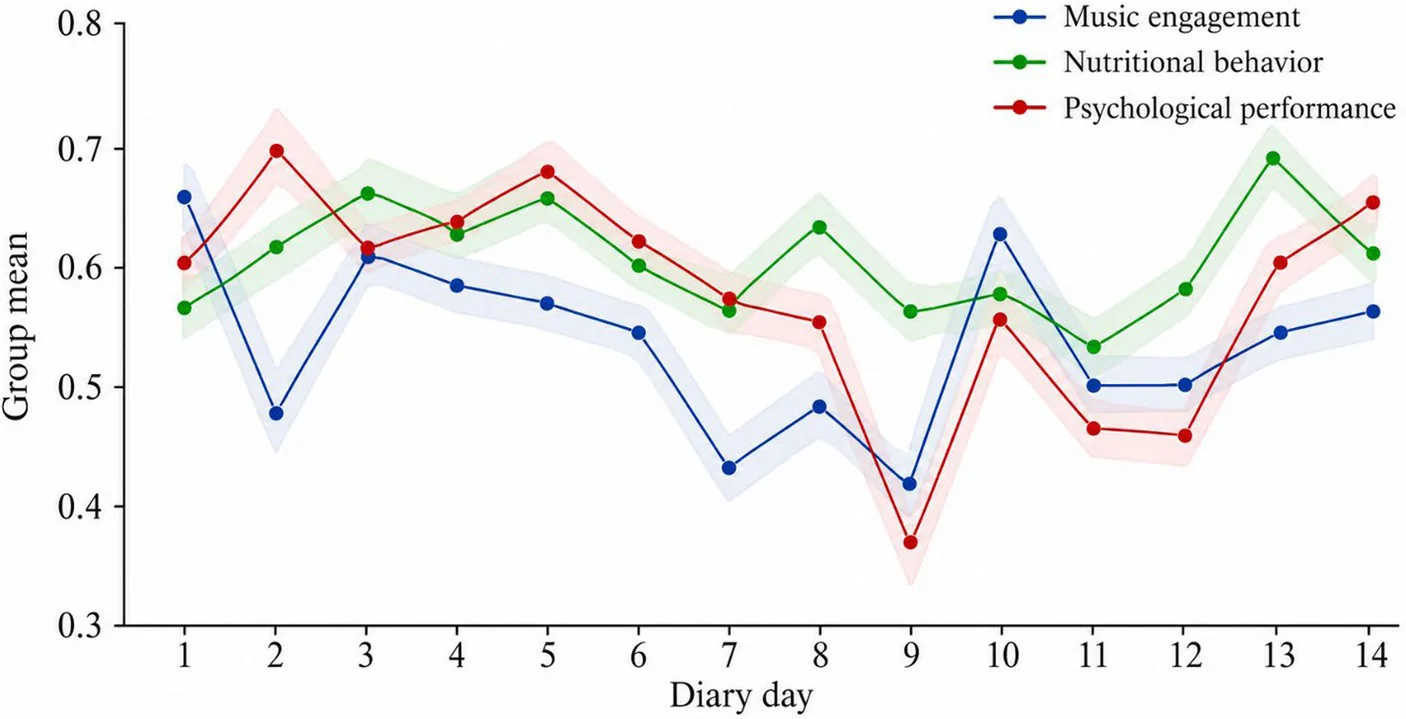
Daily group-mean trajectories. Mean music-engagement, nutritional-behavior, and psychological-performance scores across the 14-day observation period. The trajectories show modest day-to-day fluctuation without a consistent upward or downward study-period trend.

**Figure 5:**
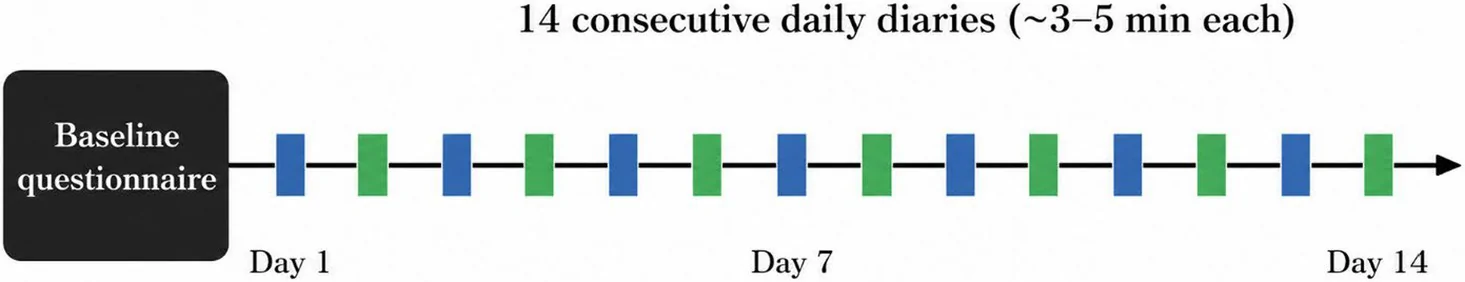
Schematic study timeline. Participants completed a baseline questionnaire followed by one digital diary per day for 14 consecutive days.

The unconditional model indicated that approximately 45% of the variance in daily psychological performance occurred between athletes and 55% occurred within athletes across days, supporting the use of a multilevel structure. The distribution and variance partition are shown in Figure 3. Daily group-mean trajectories of music engagement, nutritional behavior, and psychological performance showed modest fluctuation without a systematic upward or downward pattern across the 14-day window (Figure 4). Within-person and between-person correlations are reported in Table 2 (Figure 5).

**Table 2:** Descriptive statistics and within-/between-person correlations of daily variables.

Variable	Mean (SD)	1	2	3	4	5
1. Music engagement	3.38 (0.91)	—	0.24**	0.31***	0.09	0.19*
2. Nutritional behavior	3.55 (0.73)	0.18**	—	0.38***	0.07	0.28**
3. Psychological performance	3.62 (0.67)	0.25***	0.30***	—	−0.15	0.41***
4. Training load	5.86 (1.42)	0.10**	0.08*	−0.12***	—	−0.23**
5. Sleep quality	3.51 (0.82)	0.14***	0.22***	0.31***	−0.18***	—

Within-person correlations are reported below the diagonal and between-person correlations above the diagonal. Daily variables are composite scale means; higher scores indicate greater exposure or better status, except training load, where higher values indicate greater load. *p < 0.05, **p < 0.01, ***p < 0.001.

### 3.2 Main effects of daily music engagement and nutritional behavior

**Figure 6:**
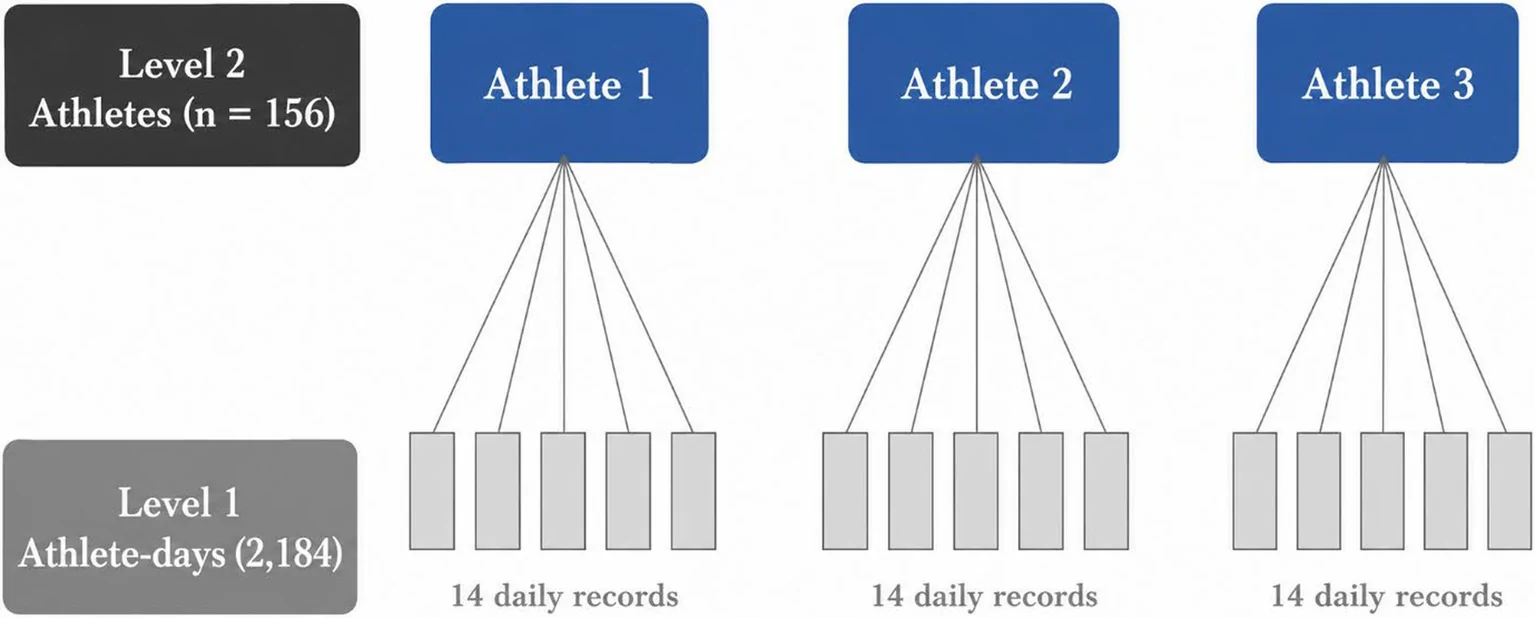
Schematic multilevel data structure. Athlete-day observations at Level 1 were nested within athletes at Level 2, with a random intercept for each athlete.

Within-person increases in daily music engagement were associated with higher psychological performance on the same day (*β* = 0.19, SE = 0.032, 95% CI = 0.13 to 0.25, *p* < 0.001) in the adjusted multilevel model (Table 3). Athletes reported better mood, focus, motivation, and readiness on days when their music engagement was higher than their own average level (Figure 6).

**Table 3:** Multilevel model of daily psychological performance.

Predictor	β (std.)	SE	95% CI	*p*
Intercept	−0.01	0.040	−0.09 to 0.07	0.80
Music engagement (within)	0.19	0.032	0.13 to 0.25	< 0.001
Nutritional behavior (within)	0.24	0.033	0.18 to 0.30	< 0.001
Music × nutrition	0.11	0.037	0.04 to 0.18	0.003
Training load	−0.08	0.026	−0.13 to −0.03	0.002
Sleep quality	0.17	0.029	0.11 to 0.23	< 0.001
Age	0.02	0.023	−0.03 to 0.06	0.42
Sex	−0.03	0.040	−0.11 to 0.05	0.46
Sport type	0.04	0.041	−0.04 to 0.12	0.33
Competitive level	0.06	0.030	0.00 to 0.12	0.048

Higher same-day psychological performance was also associated with within-person increases in daily nutritional behavior, independently of music engagement (*β* = 0.24, SE = 0.033, 95% CI = 0.18 to 0.30, *p* < 0.001). This standardized association was slightly larger than that observed for music engagement. Both associations remained statistically significant after adjustment for training load, sleep quality, sport type, age, sex, and competitive level (Table 3), indicating that they were not explained by these measured covariates.

The reported estimates are based on 2,184 athlete-day observations nested within 156 athletes. Continuous predictors were standardized, and daily music engagement and nutritional behavior were person-mean-centered to isolate within-person associations. *β* = standardized regression coefficient; SE = standard error; CI = confidence interval. Random-intercept variance = 0.45; residual variance = 0.55; marginal R^2^ = 0.18; conditional R^2^ = 0.48.

### 3.3 Music × nutrition interaction

**Figure 7:**
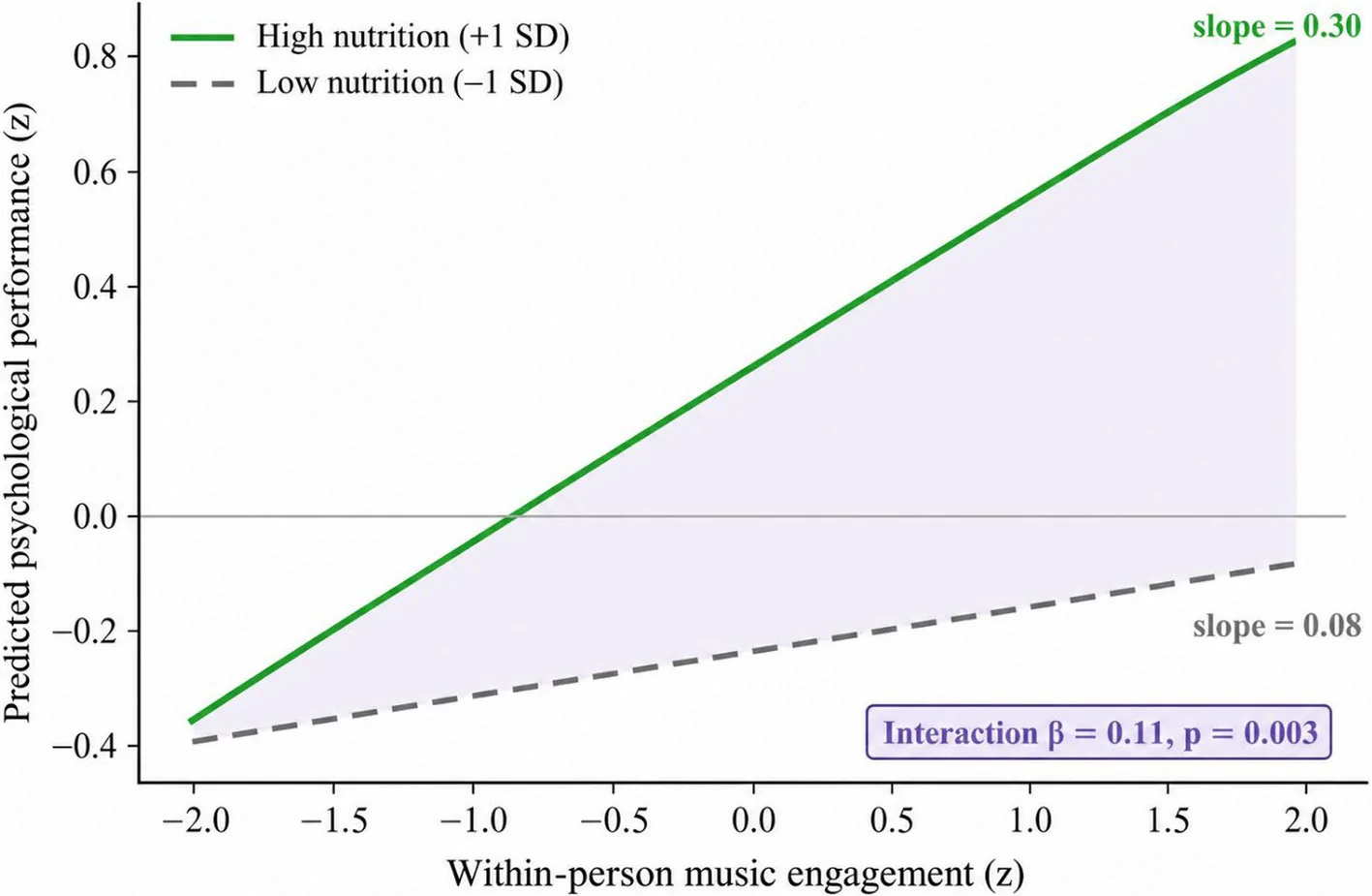
Model-estimated music × nutrition interaction. Simple-slope decomposition of the interaction (*β* = 0.11, *p* = 0.003) shows a stronger positive association between music engagement and psychological performance at relatively high nutritional behavior than at relatively low nutritional behavior.

The primary interaction analysis examined whether daily nutritional behavior modified the within-person association between music engagement and psychological performance. The music × nutrition interaction was positive and statistically significant (*β* = 0.11, SE = 0.037, 95% CI = 0.04 to 0.18, *p* = 0.003). Simple-slope decomposition indicated that the association between music engagement and psychological performance was stronger on days with relatively favorable nutritional behavior (slope ≈ 0.30) than on days with relatively unfavorable nutritional behavior (slope ≈ 0.08). The highest model-predicted psychological-performance scores occurred when both music engagement and nutritional behavior were relatively high. Adding the interaction term improved model fit relative to the main-effects-only model, with reductions of 8.9 points in the Akaike information criterion and 4.6 points in the Bayesian information criterion (Figure 7).

### 3.4 Sensitivity and robustness

The interaction estimate was examined across several alternative model specifications. After competition days were excluded, the interaction remained statistically significant (*β* = 0.10, SE = 0.039, 95% CI = 0.02 to 0.18, *p* = 0.011). A model including previous-day psychological performance produced a similar estimate (*β* = 0.09, SE = 0.038, 95% CI = 0.02 to 0.16, *p* = 0.018). Using the readiness score instead of the composite psychological-performance score also produced a positive interaction (*β* = 0.12, SE = 0.041, 95% CI = 0.04 to 0.20, *p* = 0.004). Allowing the music-engagement slope to vary randomly did not materially alter the estimate (*β* = 0.10, SE = 0.038, 95% CI = 0.03 to 0.18, *p* = 0.007). These analyses indicate consistency across the reported model specifications.

## 4 Discussion

### 4.1 Principal findings

This 14-day naturalistic diary study yielded three principal findings. First, on days when athletes reported greater music engagement than their own usual level, they also reported higher psychological performance. Second, more favorable daily nutritional behavior was independently associated with higher psychological performance, with a slightly larger standardized coefficient than music engagement. Third, a positive music × nutrition interaction was observed: the association between music engagement and psychological performance was stronger on days characterized by more favorable nutritional behavior. These findings represent day-to-day covariation within athletes after adjustment for measured covariates; they do not demonstrate that either behavior caused changes in psychological performance.

### 4.2 Interpretation in context

The positive music association is consistent with evidence linking music in exercise and sport to affective responses (10), motivation (5), perceived exertion (7), attentional regulation (6), and selected performance outcomes (8). The nutritional association is consistent with literature emphasizing the relevance of dietary quality (17), energy and macronutrient availability (15), hydration (14), and recovery (16) for athlete functioning (13). The present findings extend these separate bodies of work by examining both behaviors repeatedly within the same athletes. Several complementary pathways could potentially contribute to the observed statistical interaction, although none were directly measured. Music engagement may coincide with affective regulation (12), arousal, attentional focus, motivation, perceived exertion, autonomic activity, and stress-related responses. Favorable nutritional behavior may concurrently support hydration (27), energy availability glucose regulation, neurotransmitter synthesis, cognitive functioning, and recovery (28). Under more favorable nutritional conditions, athletes may be better positioned to experience the motivational or emotion-regulating value associated with music; conversely, hunger, dehydration, fatigue, or unstable energy availability may coincide with a weaker association. Perceptions of routine, self-regulation, control, and preparedness may also contribute. These neurophysiological, neuroendocrine, and psychological explanations remain hypotheses because the relevant biological and behavioral mediators were not measured, and the observational design does not establish causal mechanisms.

### 4.3 Strengths

The study has several methodological strengths. The intensive longitudinal design captured repeated observations in athletes’ usual training and recovery environments and permitted stable between-athlete differences to be separated from day-to-day within-athlete variation. Person-mean centering and multilevel modeling were appropriate for this analytical objective (26). The final dataset contained 2,184 athlete-day observations from 156 athletes. However, the number of repeated observations should not be described as establishing high statistical power, because precision for multilevel interaction effects depends on the number of athletes, cluster structure, intraclass correlation, effect size, and random-effects specification.

### 4.4 Limitations

Several limitations should be considered. First, the observational and same-day concurrent design precludes causal inference and does not establish temporal precedence; athletes experiencing better psychological states may be more likely to engage with music or report more favorable nutritional behavior. Second, music engagement, nutritional behavior, covariates, and psychological performance were assessed using brief self-report diary items, creating potential recall error, social-desirability bias, and common-method variance. Shared measurement procedures may have inflated associations, and the within-person psychometric properties of the author-developed composites require fuller evaluation. Third, adjustment for observed covariates cannot exclude residual confounding by daily stress, illness, social context, competition outcome, caffeine use, energy availability, or recovery status. Fourth, participant attrition and diary-level missingness may have introduced selection bias. The MCAR or MAR mechanism cannot be established from observed data (29, 30), and complete-case analysis may reduce precision or produce biased estimates when missingness is systematic (24). Fifth, convenience sampling from university teams and sports clubs limits generalizability to elite, professional, older, and culturally diverse athlete populations. Sixth, the 14-day period captured short-term variation but not longer-term, cumulative, or seasonal relationships. Finally, mechanistic biomarkers and objective measures of music exposure, dietary intake, training, sleep, and recovery were not collected.

### 4.5 Implications and future directions

If replicated and subsequently supported by experimental evidence, the observed interaction may have implications for athlete-support research. The present findings justify evaluating combined music- and nutrition-focused strategies but do not show that such strategies are more effective than approaches addressing either behavior alone. Future studies should use ecological momentary assessment with multiple assessments per day to clarify temporal order and bidirectional relationships. Nutritional exposure should be assessed using repeated 24-h recalls, weighed food records, time-stamped food photographs, hydration measures, and indicators of energy availability (31, 32). Wearable technologies could provide complementary measures of training load (33), sleep, heart-rate variability, physical activity, and recovery (34). Prospective factorial experiments should manipulate music and nutritional conditions independently to estimate their main and interactive causal effects. Mechanistic studies should assess perceived exertion, affect, cognitive performance, glucose availability, hydration, autonomic regulation, and stress-related neuroendocrine markers (35). Replication is also needed in larger samples of elite, professional, female, older, and culturally diverse athletes.

## 5 Conclusion

This study examined the independent and interactive associations of daily music engagement and nutritional behavior with psychological performance in competitive athletes. Across the reported 2,184 athlete-day observations from 156 athletes, higher-than-usual music engagement was associated with higher same-day psychological performance (*β* = 0.19), and more favorable daily nutritional behavior showed an independent positive association (*β* = 0.24). A positive music × nutrition interaction was also observed (*β* = 0.11, *p* = 0.003), indicating that the association between music engagement and psychological performance was stronger on days characterized by more favorable nutritional behavior. These within-person associations remained after adjustment for training load, sleep quality, demographic characteristics, and sport-related covariates. The observational and concurrent design does not establish temporal order, causality, intervention effectiveness, or biological mechanisms. The findings should therefore be interpreted as evidence of day-to-day covariation and as a basis for confirmatory longitudinal and experimental research (39, 40).

## Data Availability

The raw data supporting the conclusions of this article will be made available by the authors, without undue reservation.
